# Vorhersage von schweren Blutungsereignissen bei Patienten mit peripherer arterieller Verschlusskrankheit: Der OAC^3^-PAD-Risikoscore

**DOI:** 10.1007/s00772-022-00881-6

**Published:** 2022-03-11

**Authors:** Christian-Alexander Behrendt, Ulrich Rother, Christian Uhl, Hartmut Goertz, Konstantinos Stavroulakis, Alexander Gombert, die Kommission PAVK und DFS der DGG e. V.

**Affiliations:** 1 Forschungsgruppe GermanVasc, Klinik und Poliklinik für Gefäßmedizin, Universitätsklinikum Hamburg-Eppendorf, Hamburg, Deutschland; 2 Universitätsklinikum Erlangen, Erlangen, Deutschland; 3 Klinik für Gefäßchirurgie und Endovaskuläre Chirurgie, Universitätsklinikum Heidelberg, Heidelberg, Deutschland; 4 Klinik für Gefäßchirurgie, Bonifatius Hospital Lingen, Lingen, Deutschland; 5 Klinik für Gefäßchirurgie, Ludwig-Maximilians-Universitätsklinikum, München, Deutschland; 6 European Vascular Center Aachen Maastricht, Klinik für Gefäßchirurgie, Uniklinik RWTH Aachen, Aachen, Deutschland

**Keywords:** PAVK, Risikovorhersage, Antithrombotika, Blutungen, Gerinnung, PAD, Risk Prediction, Antithrombotics, Bleeding, Coagulation

## Abstract

Obwohl Patient:innen mit einer peripheren arteriellen Verschlusskrankheit (PAVK) aufgrund ihres Komorbiditäts- und Risikoprofils ein insgesamt erhöhtes Blutungsrisiko aufweisen, standen bisher keine validierten Werkzeuge zur Vorhersage des Blutungsrisikos zur Verfügung. Erschwerend kommt hinzu, dass viele randomisierte und kontrollierte Studien zu antithrombotischen Therapien Patient:innen mit erhöhtem Blutungsrisiko ausgeschlossen haben. Anhand von Routinedaten der Krankenkasse wurde mittels Verfahren des maschinellen Lernens ein pragmatisches Vorhersagemodell entwickelt und intern validiert. Mit dem OAC^3^-PAD-Risikoscore wurden acht Variablen identifiziert, die das Risiko von schweren Blutungsereignissen innerhalb eines Jahres nach stationärer Behandlung der PAVK vorhersagen können. Dieser Risikoscore kann dabei helfen, eine patientenzentrierte Risiko-Nutzen-Abwägung durchzuführen, um das maximale Potenzial aus den verfügbaren antithrombotischen Therapiestrategien zu schöpfen.

## Abstract (en)

Although patients with peripheral arterial disease (PAD) are at a high risk of major bleeding owing to their comorbidity and risk profile, no validated tools exist to predict bleeding risk. To make matters worse, several randomized and controlled trials have excluded patients who are at a high risk of bleeding. Using routine health insurance claims data and machine learning methods, a pragmatic prediction model was developed and internally validated. The OAC^3^-PAD risk score identified eight variables that can predict major bleeding events within 1 year of inpatient treatment for PAD. This risk score can help to carry out a tailored patient-centered risk–benefit assessment in order to obtain the maximum potential from available antithrombotic treatment strategies.

## Vorhersage von schweren Blutungsereignissen bei Patienten mit peripherer arterieller Verschlusskrankheit

Kaum ein anderes Thema – ausgenommen vielleicht COVID-19 oder die Paclitaxel-Debatte – erhält aktuell so viel Aufmerksamkeit durch die gefäßmedizinische Community, wie die optimale Arzneimitteltherapie der peripheren arteriellen Verschlusskrankheit (PAVK). Neben der lipidsenkenden, antihypertensiven und antidiabetischen Therapie steht außer Zweifel, dass Thrombozytenaggregationshemmer in symptomatischen Krankheitsstadien sowohl das langfristige Gesamtüberleben als auch die Amputationsraten substanziell verbessern können [1, 7, 11, 18]. Es ist dabei allgemein bekannt, dass Patient:innen mit einer PAVK, unter allen atherosklerotischen Entitäten, sowohl zu der Gruppe mit dem ausgeprägtesten Risikoprofil als auch besonders schlechten Langzeitergebnissen gehören. Etwa 80 % der invasiv revaskularisierten PAVK-Patient:innen in Deutschland gaben an, jemals aktiv geraucht zu haben, 44 % sogar aktiv zum Zeitpunkt der stationären Behandlung [14]. Die 10-Jahres-Inzidenz von Lungenkrebs nach erstmaliger Indexbehandlung der PAVK beträgt 7 % bei Männern und 4 % bei Frauen, was den negativen Einfluss des Rauchens in dieser Population zusätzlich unterstreicht [13]. Bis zu 30 % haben nach den gültigen Kriterien der Weltgesundheitsorganisation (WHO) eine Adipositasdiagnose und 20 % leiden unter einer laborchemisch nachweisbaren Dyslipidämie [14, 16]. Ein Viertel der Patient:innen ist bereits klinisch herzinsuffizient und das konsekutive Risiko für Tod oder Amputation beträgt letztendlich zwischen 9 und 48 % bei Patient:innen mit Claudicatio intermittens bzw. 45 und 88 % bei Patient:innen mit chronischer extremitätengefährdender Ischämie [15, 16]. Zusammenfassend illustrieren diese bemerkenswerten Fakten, wie wichtig die Vermeidung von thromboembolischen Ereignissen in allen Gefäßregionen ist, was zusätzlich durch die hohe Prävalenz von kardialen Rhythmusstörungen (25 %) unterstrichen wird [14, 16]. Es erscheint daher naheliegend, die Intensität der antithrombotischen Therapie dementsprechend so weit auszureizen, bis deren Komplikationsrisiko eine weitere Intensivierung nicht mehr zulässt. Allerdings muss vor diesem Hintergrund auch angemerkt werden, dass etwa 30 % der Patient:innen eine chronische Niereninsuffizienz aufweisen und ungefähr 10–20 % bereits dementiell erkrankt und daher sturzgefährdet sind [14, 16]. Das Thema Frailty (Gebrechlichkeit), Multimorbidität und Polypharmazie erfährt gegenwärtig vor dem Hintergrund der demografischen Entwicklungen zunehmende Bedeutung.

Verschiedene randomisierte kontrollierte Studien (RCT) zu den antithrombotischen Therapiestrategien bei Patient:innen mit PAVK haben nachgewiesen, dass eine verlängerte oder intensivierte gerinnungswirksame Therapie mit höheren Blutungsraten assoziiert werden konnte. Die überwiegende Mehrheit der Studien hat dabei designbedingt Patient:innen mit erhöhten Blutungsrisiken ausgeschlossen und die Leitlinienempfehlungen beschränken sich daher primär auf diejenigen Patient:innen mit hohem Thromboembolierisiko ohne erhöhtes Blutungsrisiko [2, 6, 9, 12, 19]. In der Kardiologie existieren zur Einschätzung des mittel- und langfristigen Blutungsrisikos etablierte Vorhersagemodelle bzw. Scores, die aufgrund der Unterschiede bei den Komorbiditätsprofilen allerdings nicht ohne Weiteres bzw. direkt auf die Zielpopulation PAVK anzuwenden sind. So wurden mit HAS-BLED und HEMORR2HAGES zwei Risikoscores für Patient:innen mit Vorhofflimmern entwickelt [10, 17]. Die Konsensusempfehlungen zu ARC-HBR gelten vor allem für Patient:innen mit perkutanen Koronarinterventionen [20]. Ähnliche Einschränkungen gelten für ATRIA, PE-CH, RIETE, mOBRI und ACCP. Lediglich der Risikoscore, der anhand des REACH-Registers entwickelt wurde, hat ambulant behandelte Patient:innen mit stabilem Verlauf und hohem Risiko für atherothrombotische Ereignisse adressiert und konnte daher eine relevante Anzahl an PAVK-Patient:innen einschließen, wenn auch nicht primär adressieren [8]. Bis heute fehlt daher ein valider Risikovorhersagescore, der das Blutungsrisiko gleichermaßen objektivierbar und pragmatisch in der stationären Versorgungsrealität abbilden kann.

**Figure 1:**
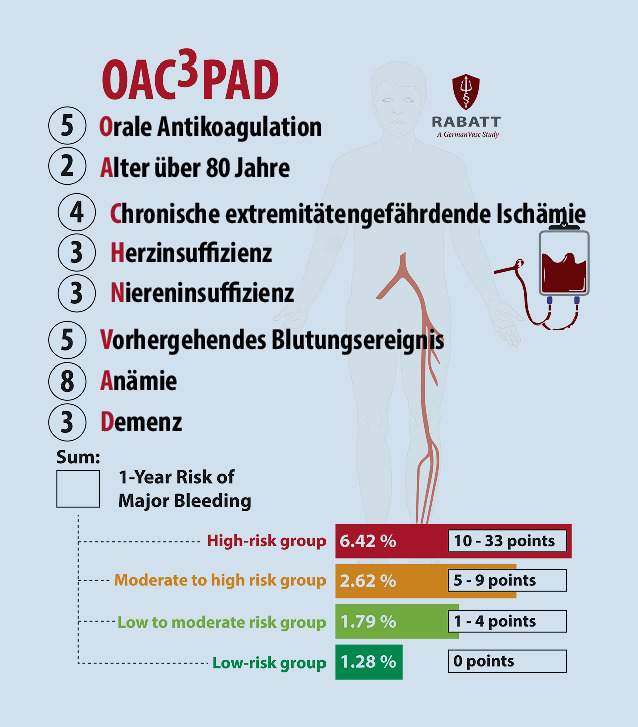

Mit dem OAC^3^-PAD (https://score.germanvasc.de) wurde durch das Leitlinienkomitee der European Society for Vascular Surgery (ESVS) zur antithrombotischen Therapie bei Gefäßkrankheiten kürzlich ein pragmatischer Score entwickelt und intern validiert, bei dem nur acht Faktoren das 1‑Jahres-Risiko für schwere stationär behandlungsbedürftige Blutungen vorhersagen konnten: orale Antikoagulation, hohes Alter über 80 Jahre, chronische extremitätengefährdende Ischämie, Herzinsuffizienz, Niereninsuffizienz, vorhergehende Blutungsereignisse, Anämie und Demenz (Abb. 1; [3]). Nach Summierung der einzelnen Gewichtungsfaktoren kann die Einteilung in eine der vier Risikogruppen von Niedrigrisiko (1,3 %) bis Hochrisiko (6,4 %) erfolgen, wobei etwa 20 % aller PAVK-Patient:innen ein hohes Risiko aufwiesen. Insgesamt erlitten etwa 2,2 % der Kohorte innerhalb eines Jahres eine schwere Blutung (4,5 % nach 3 Jahren). Darunter ca. 15 % intrakraniell und etwa 60 % gastrointestinal, was die Bedeutung der Protonenpumpenhemmer unterstreicht. Für die Entwicklung des OAC^3^-PAD wurden faktisch anonymisierte Routinedaten der BARMER verwendet und Verfahren aus dem Bereich des maschinellen Lernens angewandt. Insgesamt sind dabei Datensätze zu 81.930 Patient:innen mit stationären Behandlungen zwischen dem 1. Januar 2010 und 31. Dezember 2018 in die Analysen eingegangen, wobei zur Identifizierung von Komorbiditäten und Indexbehandlungen auch auf zurückliegende Daten bis 2005 zugegriffen wurde.

Neben dem hohen Alter, Niereninsuffizienz, vorhergehenden Blutungsereignissen und Anämie, die in zahlreichen Risikovorhersagemodellen eine Rolle spielen, konnten Demenz, Herzinsuffizienz und fortgeschrittene PAVK-Stadien erstmals als zentrale Prädiktoren identifiziert werden.

Neben der Heterogenität von Variablen und deren Definitionen in den früheren kardiologischen Blutungsscores wurde offensichtlich, dass auch die zentralen Endpunktdefinitionen zwischen den Studien und Registern variierten. Obwohl internationale Konsensusempfehlungen zu PAVK-Registern die Erhebungen von Blutungsereignissen empfohlen haben, wurden dort keine eindeutigen Aufgreifkriterien für Blutungsereignisse benannt [4, 5]. Dementsprechend eingeschränkt bleibt die Vergleichbarkeit dieses wichtigen Sicherheitsendpunkts. Der OAC^3^-PAD Risikoscore nutzt dabei eine Definition, die am ehesten mit der Definition der International Society on Thrombosis and Haemostasis (ISTH) für Majorblutung vergleichbar ist [21]. Unabhängig von der konkreten Definition der Endpunkte ist allerdings insbesondere in Registererhebungen davon auszugehen, dass eine Untererfassung von Ereignissen immanent ist, da Patient:innen bei intrakraniellen und gastrointestinalen Blutungen vermutlich eher in anderen Abteilungen bzw. Einrichtungen behandelt werden oder an infausten Ereignissen versterben. Dieses weitverbreitete Problem wird auch als Non-Registration-Bias bezeichnet und ist bisher weitgehend ungelöst; insbesondere solange die Rate an postmortalen Autopsien in Deutschland so niedrig ist.

Externe Validierungsprojekte zum OAC^3^-PAD wurden bereits mit populationsbasierten Daten in Schweden, England, Frankreich und den Vereinigten Staaten initiiert. Es erscheint zudem sinnvoll und machbar, ein von der Deutschen Gesellschaft für Gefäßchirurgie und Gefäßmedizin (DGG) e. V. getragenes Validierungsprojekt mit prospektiv erhobenen Primärdaten zu initiieren.

Grundsätzlich erscheint es ratsam, das Blutungsrisiko unserer Patient:innen individuell zu kalkulieren und in alle Erwägungen zur antithrombotischen Therapie einzubeziehen. Das kann sowohl der Wechsel auf ein anderes Monopräparat, die (temporäre) Einleitung einer dualen Thrombozytenaggregationshemmung oder auch eine neue Dual-pathway-Therapie sein.

Nicht selten finden sich sowohl eine orale Antikoagulation in voller Dosierung als auch ein oder sogar zwei Thrombozytenaggregationshemmer in den Entlassungsempfehlungen, was unsere Bemühungen zur Erhaltung der peripheren Durchblutung unterstreicht. Eine derart aggressive antithrombotische Triple-Therapie ist allerdings, bis auf wenige Ausnahmen, nicht sicher bzw. gerechtfertigt. Nicht immer erhalten die behandelnden Gefäßmediziner:innen allerdings auch Kenntnis über stattgehabte Blutungsereignisse im Verlauf nach der Krankenhausentlassung. Die generelle Verbesserung der Awareness und Implementierung einer risikoadaptierten Strategie erscheint daher grundsätzlich wünschenswert, um das volle Potenzial aus den verfügbaren antithrombotischen Strategien herauszuholen.

## Fazit für die Praxis

- Patient:innen mit einer peripheren arteriellen Verschlusskrankheit haben aufgrund ihrer Komorbiditäten ein gegenüber der Normalbevölkerung erhöhtes Blutungsrisiko.
- Jede antithrombotische Therapie sollte unter Abwägung des Blutungs- und Thrombembolierisikos festgelegt werden; der OAC^3^-PAD-Risikoscore kann dabei helfen, dieses Risiko patientenindividuell vorherzusagen (https://score.germanvasc.de).
- Bisher standen vor allem Risikovorhersagemodelle für Blutungen bei Patient:innen mit kardialen Erkrankungen zur Verfügung.
- Acht Variablen sagen unabhängig das Risiko schwerer Blutungen innerhalb eines Jahres vorher: orale Antikoagulation, Alter über 80 Jahre, chronische extremitätengefährdende Ischämie, Herzinsuffizienz, Niereninsuffizienz, frühere Blutungen, Anämie, Demenz.
- Insgesamt erlitten etwa 2,2 % der Patient:innen mit PAVK innerhalb eines Jahres eine schwere Blutung (4,5 % nach 3 Jahren). Darunter ca. 15 % intrakraniell und etwa 60 % gastrointestinal, was die Bedeutung der Protonenpumpenhemmer unterstreicht.
